# Regulation of Apoptosis during Flavivirus Infection

**DOI:** 10.3390/v9090243

**Published:** 2017-08-28

**Authors:** Toru Okamoto, Tatsuya Suzuki, Shinji Kusakabe, Makoto Tokunaga, Junki Hirano, Yuka Miyata, Yoshiharu Matsuura

**Affiliations:** Department of Molecular Virology, Research Institute for Microbial Diseases, Osaka University, Osaka 565-0871, Japan; tsuzuki@biken.osaka-u.ac.jp (T.S.); shinji@biken.osaka-u.ac.jp (S.K.); mtoku@biken.osaka-u.ac.jp (M.T.); kamati1101@biken.osaka-u.ac.jp (J.H.); yuppi_ramune0309@yahoo.co.jp (Y.Mi.); matsuura@biken.osaka-u.ac.jp (Y.Ma.)

**Keywords:** apoptosis, B-cell lymphoma 2 (BCL2), flavivirus, dengue virus, Japanese encephalitis virus, West Nile virus

## Abstract

Apoptosis is a type of programmed cell death that regulates cellular homeostasis by removing damaged or unnecessary cells. Its importance in host defenses is highlighted by the observation that many viruses evade, obstruct, or subvert apoptosis, thereby blunting the host immune response. Infection with Flaviviruses such as Japanese encephalitis virus (JEV), Dengue virus (DENV) and West Nile virus (WNV) has been shown to activate several signaling pathways such as endoplasmic reticulum (ER)-stress and AKT/PI3K pathway, resulting in activation or suppression of apoptosis in virus-infected cells. On the other hands, expression of some viral proteins induces or protects apoptosis. There is a discrepancy between induction and suppression of apoptosis during flavivirus infection because the experimental situation may be different, and strong links between apoptosis and other types of cell death such as necrosis may make it more difficult. In this paper, we review the effects of apoptosis on viral propagation and pathogenesis during infection with flaviviruses.

## 1. Introduction

### 1.1. Overview of Apoptosis Signaling

**Figure 1:**
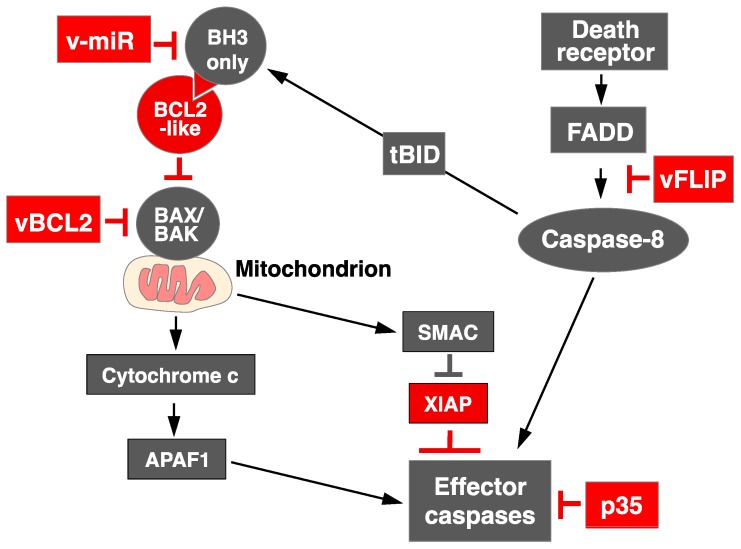
Pathways of apoptosis. Prosurvival proteins (BCL2, BCLW, BCLX_L_, MCL1, and A1) sequester BH3-only proteins to prevent the activation of BAX and BAK under normal conditions. In the presence of apoptotic stimuli, such as the deprivation of cytokines or cellular damage, BH3-only proteins are activated and displace the prosurvival proteins from their interaction with BAX and BAK, disrupting the mitochondrial outer membrane and thus releasing cytochrome c, second mitochondria-derived activator of caspases (SMAC), and so on. The cytochrome c released interacts with apoptotic protease-activating factor 1 (APAF1) to activate effector caspases, including caspase 9. At same time, SMAC blocks the caspase inhibitor X-linked inhibitor of apoptosis proteins (XIAP). Death receptors, such as FAS/CD95 or tumor necrosis factor receptors (TNFR), are activated by their ligands (FASL or TNF, respectively) and then recruit FAS-associated death domain protein (FADD) to cytoplasmic domains of receptors, resulting in the activation of caspase 8 and effector caspases. Activated caspase 8 cleaves BID to generate truncated BID (tBID), inducing mitochondria-mediated apoptosis. Some DNA viruses encode several genes to control apoptosis (shown in red). The black arrows indicated signaling pathways and T bars shows suppression of activity of indicated proteins.

Apoptosis, or programmed cell death, is an evolutionarily conserved process essential for the removal of damaged, infected or excess amounts of cells [1,2]. It is required for normal development, tissue homeostasis, and countering infection. Whether a cell lives or dies in response to diverse developmental cues or cellular stresses is largely determined by the interactions of three members of this protein family, Bcl-2 homology 3 (BH3)-only proteins, BCL2 proteins and Bcl-2-associated X protein (BAX)/Bcl-2 homologous antagonist/killer (BAK) effectors [1]. To promote apoptosis, the BH3-only proteins (e.g., Bcl-2 interacting mediator (BIM), Bcl-2 associated death promoter (BAD), NADPH oxidase activator (NOXA) sense cellular damage, but the critical downstream mediators of apoptosis are BAX and BAK, and their combined absence abolishes most apoptotic responses [1,2]. When activated, BAX and BAK permeabilize the outer mitochondrial membrane, releasing proapoptogenic factors, such as cytochrome c, which then promote the activation of the cysteine proteases (caspases) that mediate cellular destruction [3]. The activation of BAX and BAK is opposed by the prosurvival proteins, including BCL2, B-cell lymphoma-extra -large (BCLXL), BCL2-like 2 protein (BCLW), myeloid cell lymphoma 1 (MCL1), and Bcl-2 related protein A1 (A1). These proteins are inactivated by the insertion of BH3 domains of the BH3-only proteins into a groove on the prosurvival proteins to allow the activation of BAX and BAK [1,4]. Activated BAX and BAK then permeabilize the outer mitochondrial membrane to release proapoptotic molecules, such as cytochrome c and second mitochondria-derived activator of caspases (SMAC). Cytochrome c interacts with apoptotic protease-activating factor 1 (APAF1) to activate the effector caspases, including caspase 9 [5]. At same time, SMAC block the caspase inhibitor, X-linked inhibitor of apoptosis proteins (XIAP). Death receptors, such as FAS/CD95 and tumor necrosis factor receptors (TNFR), are activated by their ligands (FASL and TNF, respectively) and then recruit the FAS-associated death domain protein (FADD) to cytoplasmic domains of receptors, resulting in the activation of caspase 8 and the effector caspases [6]. To link mitochondria-mediated apoptosis and death-receptor-mediated apoptosis, caspase 8 is activated by the death receptors and cleaves BH3 interacting domain death agonist (BID) to generate truncated BID, inducing mitochondria-mediated apoptosis [7,8] (Figure 1).

### 1.2. Application of Mitochondria Mediated Apoptosis to Cancer Therapy

Small-molecule inhibitors ABT-737 [9] (or ABT-263 (navitoclax), an orally available clinical derivative of ABT-737 [10]), ABT-199 (venetoclax) [11], and A-1331852 [12] have been developed as cancer therapeutics. Whereas ABT-737/ABT-263 targets BCL2, BCLW, and BCLX_L_, ABT-199 and A-1331852 target BCL2 and BCLX_L_, respectively. The U.S. Food and Drug Administration has approved ABT-199 for the treatment of 17-p-deleted chronic lymphocytic leukemia (CLL) [13]. It should be noted that the induction of cell death by targeting BCL2 proteins is a promising therapeutic strategy based on the removal of unnecessary cells.

### 1.3. Apoptosis Regulation by RNA Virus: Lessons from DNA Virus

The consequences of effects of apoptosis during infection with RNA virus remain unclear. However, apart from RNA virus, DNA viruses have evolved a capacity to control cell death [14]. For example, adenovirus, Karposi’s sarcoma-associated herpesvirus (KSHV), Epstein-Barr virus (EBV), and murine gamma-herpesvirus 68 (γHV68) encode homologues of mammalian BCL2: ADE1B19K, KSHV vBCL2, BHRF-1, and HV68 M11, respectively. These homologues act as prosurvival proteins by inhibiting the activation of BAX and BAK. Some viruses also encode microRNAs that target proapoptotic host proteins [15,16,17]. For example, EBV encodes a microRNA, miR-BART5, that targets BH3-only proteins, including p53 upregulated modulator of apoptosis (PUMA) [18]. Furthermore, the majority of poxviruses encode a viral BCL2, which has comparable functions to the BCL2 protein family, despite of the lack of sequence similarity [19,20]. Importantly, these viral proteins are also associated with viral virulence. The p35 protein encoded by the baculoviruses is a viral inhibitor of apoptosis that inhibits a broad range of caspases [21]. Furthermore, virus-encoded Fas-associated death domain-like interleukin-1β (IL-1β)-converting enzyme inhibitory proteins (vFLIP) inhibit the activation of caspase 8 by targeting a complex of FADD and caspase 8 [22,23,24]. The CrmB protein encoded by orthopoxviruses is a soluble protein that tightly binds TNF to inhibit TNF/TNFR-mediated apoptosis [25]. These data suggest that DNA viruses have acquired strategies to inhibit cell death in order to prolong infection and enhance the production of progeny viruses.

### 1.4. Life Cycle of Flavivirus Infection

**Figure 2:**
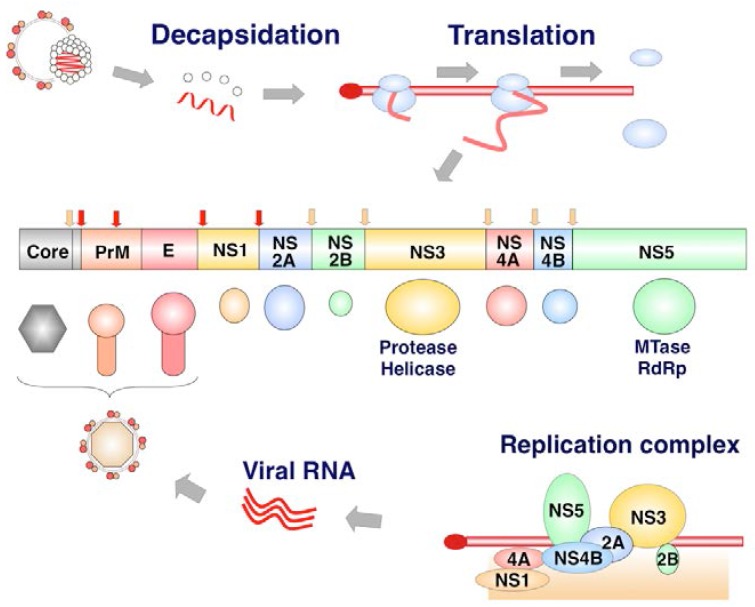
Structure of the flavivirus polyprotein. After decapsidation, the *Flavivirus* genome acts as an mRNA and is directly translated to a polyprotein in a Cap-dependent manner. The polyprotein is cleaved by host signal peptidase (SP) and viral protease (NS3). Core, prM, and E are components of the viral particles. Nonstructural (NS) proteins form a complex on the ER membrane and produce viral RNA. NS5 has methyltransferase (MTase) and RNA-dependent RNA polymerase (RdRp) activities. Red arrow shows host proteases cleave viral proteins while yellow arrow shows cleavages of viral proteins by viral proteases.

The family *Flaviviridae* contains viruses that are global human health concerns, including *Dengue virus* (DENV), *West Nile virus* (WNV), *Japanese encephalitis virus* (JEV), and *Zika virus* (ZIKV) [26,27]. The viruses of the family *Flaviviridae* have a positive-sense single-stranded RNA genome. The viral RNA is translated to a single polyprotein (of about 3000 amino acids) that is cleaved by host proteases and specific viral proteases, producing at least 10 viral proteins (Figure 2).

**Figure 3:**
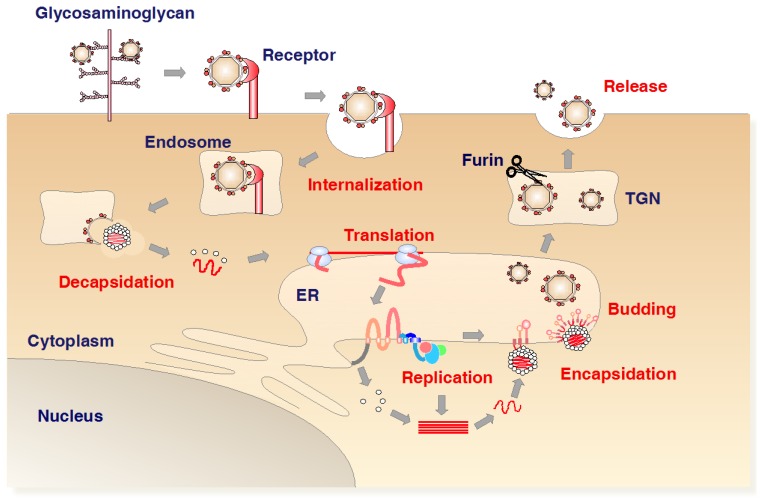
Life cycle of the flaviviruses. The virus first binds to glycosaminoglycan (GAG) and then to specific receptors, and enters the cell by endocytosis. The *Flavivirus* genome is a single-stranded positive-sense RNA molecule that acts directly as an mRNA, which is translated into a polyprotein (Figure 1). Nonstructural proteins form a replication complex that replicates the viral RNA on the ER. Capsid protein molecules incorporate the viral RNA, and the immature virus buds from the ER together with prM and E. The immature protein is processed in the trans-Golgi network, with the removal of prM by furin. Mature virus is subsequently released by exocytosis.

The core protein is the first protein to be translated from the viral genome and cleaved by the viral NS2B/3 protease and then the host signal peptidase. The core protein forms a dimer and the viral capsid. The precursor membrane (PrM) and envelope (E) are translated into the ER. The PreM assists the proper folding of E, and plays a role in shielding the fusion peptide of E protein. The E protein is a protein representing on the surface of virions, and is important for receptor binding and membrane fusion. Seven nonstructural proteins are components of the viral replication complex (VRC) [28,29]. The NS1 glycoprotein is translated into the ER, and the intracellular dimer form of NS1 plays roles in viral RNA replication, where the NS1 hexamer is secreted from mammalian cells to play a role in evasion of humoral immune responses. The NS2B and NS3 (NS2B-3) is a serine protease. The NS5 possesses activity of methyltransferase (MTase) and RNA-dependent RNA polymerase (RdRp). The life cycle of the flaviviruses is summarized in Figure 3.

Viral particles primarily interact with glycosaminoglycans, then bind to specific receptors, and are internalized into cells via endocytosis. Under the acidic conditions in the endosome, the capsid releases into cytoplasm after fusion of the viral membrane with the cellular membrane, and the viral RNA is directly translated into a precursor polyprotein. The VRC is formed on the endoplasmic reticulum (ER). The immature viral particles bearing prM and E bud into ER rumen after nucleocapsid formation with viral RNA. PrM is cleaved in the trans-Golgi network by the cellular protease furin to form the mature particles. The mature virions are released by exocytosis.

## 2. Apoptosis during Flavivirus-Infection

### 2.1. Pro-Survival and Pro Apoptotic Activity of Viral Proteins

**Figure 4:**
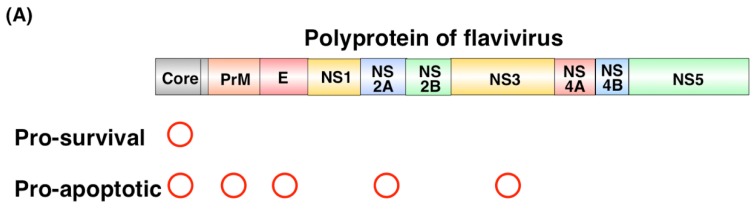

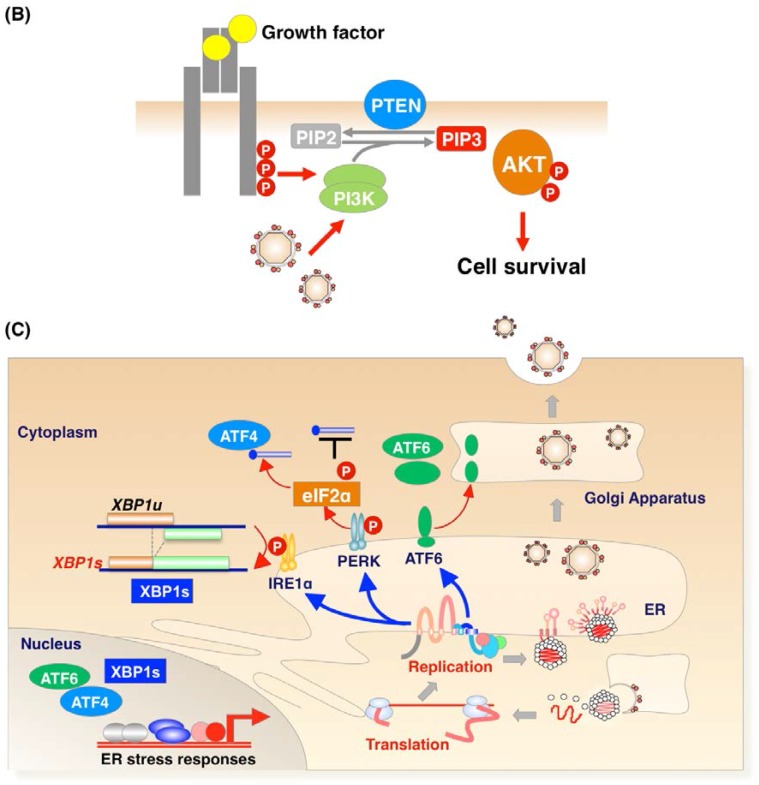
*Flavivirus* infection regulates apoptosis. (**A**) Regulation of viral proteins. Several viral proteins function as proapoptotic proteins; (**B**) AKT/PI3K signaling pathway. In general, growth factors, such as insulin, bind to their receptors to induce receptor phosphorylation, leading to the activation of PI3K, which stimulates the phosphorylation of AKT. Phosphorylation of AKT promotes cellular events, including the suppression of proapoptotic proteins or the promotion of cell survival. Core protein of WNV or infection by JEV or DENV activates the phosphorylation of AKT by activating PI3K to block caspase-dependent apoptosis; (**C**) ER stress induction. The accumulation of unfolded proteins induces ER stress, which stimulates three molecules, IRE1α, PERK, and ATF6. IRE1α is a serine/threonine protein kinase with endonuclease activity. ER stress induces the autophosphorylation of IRE1α to catalyze the excision of XBP1 mRNA. The phosphorylation of PERK, a protein kinase, is induced by ER stress and p-PERK phosphorylates eIF2α to suppress protein translation and to induce the expression of ATF4. ATF6 is also activated by ER stress, translocated to the Golgi, and activated by cleavage of the site-2 proteinase. XBP1, ATF4, and ATF6 are transcription factors to result in response to ER stress. Thus, *Flavivirus* infection stimulates ER stress in many ways.

Viral proteins have been shown to regulate apoptosis, as summarized in Table 1. Netsawang et al. showed that nuclear localization of DENV capsid protein is required for its interaction with Fas death domain associated protein xx (DAXX) and the induction of apoptosis [30]. The WNV capsid protein also induces apoptosis through the interaction with importin-α and the phosphorylation by protein kinase C [31]. These results suggest that the nuclear localization of the capsid protein facilitates caspase-9-dependent apoptosis. In contrast, the WNV capsid protein stimulates the phosphorylation of AKT to suppress the activation of caspases 3 and 8 [32]. The ectodomain of the flavivirus M protein also induces apoptosis, but the overexpression of BCL2 suppresses the cell death induced by the M protein [33]. The E protein, but not the NS2B-3, of *Langat virus* (another flavivirus) also induces apoptosis [34]. Injection of recombinant DENV-E protein domain III suppressed megakaryopoiesis through activation of apoptosis in its progenitors [35]. A mutation in the WNV NS2A protein, converting alanine 30 to proline (A30P), attenuated viral virulence by an unknown mechanism [36]. The propagation of the mutant A30P virus was similar to that of the wild-type (WT) virus, but the A30P virus showed less severe cytopathic effects. The number of terminal deoxynucleotidyl transferase dUTP nick end labeling (TUNEL)-positive cells was significantly reduced by the infection with the mutant WNV [37], suggesting that NS2A is involved in WNV-induced apoptosis and pathogenesis. The protease activity of NS3 in JEV, DENV, and WNV induces apoptosis through the activation of caspase 3 or caspase 8. NS2B, a cofactor of NS3, is required for the induction of NS3-induced apoptosis [38,39,40,41] (Figure 4A).

### 2.2. Pro-Apoptotic or Pro-Survival Activity during Viral Infection

It is important for us to understand the consequences of regulation of apoptosis during viral infection, and not only the viral protein itself. *Flavivirus* infection has been shown to induce or protect apoptosis as summarized in Table 2. DENV isolated from human patients induced apoptosis in the mouse neuroblastoma cell line, Neuro 2a [42], endothelial cells [43,44], liver cancer cell lines, such as HepG2 [45,46] and Hep3B [47], and human monocyte-derived dendritic cells (Mo-DC) [48,49]. DENV also induced TP53 expression and mitochondrial permeabilization through TP53 [50]. WNV also induced BAX-dependent apoptosis in Neuro 2a cells and K562 cells [51]. Furthermore, apoptosis was induced in neurons derived from embryonic stem (ES) cells during WNV infection [52]. The AKT/PI3K pathway controls cell growth, survival, and energy consumption (Figure 4B). Lee et al. showed that infection with JEV or DENV upregulated the phosphorylation of AKT to activate PI3K/AKT signaling, resulting in the blockage of caspase-mediated cell death [53]. Treatment with LY294002, a PI3K inhibitor, accelerated virus-induced apoptosis [53]. The accumulation of unfolded proteins in the ER triggers the unfolded protein stress response, leading to ER stress [54]. ER stress induces the upregulation of chaperones to refold the unfolded proteins, the ER-associated degradation of any unfolded proteins, and the death of unnecessary cells. *Flavivirus* infection stimulates the ER stress response. JEV, WNV, and DENV infections activate inositol-requiring protein 1α (IRE1α), protein kinase RNA-like ER kinase (PERK), or activating transcription factor 6 (ATF6) [46,55,56,57,58]. The activation of IRE1α, PERK1, and ATF6 causes the mRNA encoding unspliced X box-binding protein 1 (XBP1u) to be processed, the expression of ATF4, and the cleavage of ATF6 in the Golgi apparatus, respectively. The ER stress induced by JEV and DENV has been shown to activate ER-associated protein degradation to protect the infected cells from cell death [59]. However, other research groups have shown that the induction of ER stress is associated with DENV pathogenesis [56] (Figure 4C). These discrepancies may be attributable to the different extent of ER stress induced in different cell lines or by different viral strains. Further research, using animal models or gene-edited mice, is required to understand the physiological consequences of the induction of ER stress by flaviviruses. WNV infection also upregulates cellular microRNA Hs_154, to induce apoptosis by targeting antiapoptotic proteins, including the coamplified CCCTC-binding factor (CTCF) and epidermal growth factor receptor (EGFR) and the overexpressed ECOP and VOPP1 proteins. The inhibition of Hs_154 inhibited apoptosis during WNV infection [60].

### 2.3. Physiological Significance of Apoptosis Signaling during Flavivirus Infection

As described above, many studies have shown that viral infection or the expression of viral proteins induces various types of apoptosis, with the activation of caspases. However, it is important to understand the physiological consequences of apoptotic regulation during viral infection and especially whether the induction or suppression of apoptosis is regulated by host factors to clear infected cells or by the virus to achieve its efficient propagation in vivo. In a mouse model, a deficiency of caspase 3 reduced the disease symptoms and mortality rate during WNV infection, whereas viral propagation was similar in the knockdown and WT mice. This suggests that the lack of caspase 3 activity impaired cell death, suppressing the viral burden and reducing neuronal injury [61]. Not only the extrinsic pathway, but also the mitochondria-mediated apoptotic pathway, leads to the activation of effector caspases, such as caspase 3. A recent study suggested that the blockage of effector caspases caused adverse effects, such as the cytosolic DNA sensor cyclic GMP-AMP synthase (cGAS)/stimulator of interferon gene (STING)-mediated induction of type I interferon (IFN) through mitochondrial DNA (mtDNA) [62,63].

The same research group also reported the functions of the TNF family of ligands, TNF-related apoptosis-inducing ligand (TRAIL) and Fas-ligand (FASL), during WNV infection. The pathogenicity of WNV was enhanced in FASL-deficient (gld) mice or TRAIL^−/−^ mice after their subcutaneous infection [64,65]. There were significantly more viral particles in the brains of the gene-deficient mice than in the WT mice, whereas viral propagation in the brain after intracranial infection with WNV did not differ between the two groups. The CD8^+^ T cells in both mouse groups showed impaired clearance of the WNV-infected cells. These findings strongly suggest that death-receptor-mediated apoptosis is required for the clearance of WNV-infected cells.

## 3. Conclusions and Future Directions

The regulation of apoptosis by RNA viruses, including flaviviruses, seems to be more complex than its regulation by DNA viruses. Lessons from the DNA viruses suggest that viruses inhibit apoptosis to achieve efficient propagation in vivo, but apoptosis seems to be induced in virus-infected cells by a host response to clear the infected cells in vitro. Recent advances in gene-editing technologies, such as CRISPR/Cas9, allow us to generate gene-modified mice, including knockout and knockin mice. Therefore, using these technologies, future studies should extend our understanding of the physiological importance of apoptosis during viral infection in vivo. In terms of antiviral therapies, apoptotic cells were detected within 4 h in CLL patients treated with ABT-199 in vitro experiments and the rapid clearance of apoptotic cells was observed at 6–24 h in vivo [66,67]. Therefore, the control of apoptosis by BH3 mimetics can efficiently remove apoptotic cells, including viral-infected cells, from the human body.

## Figures and Tables

**Table 1:** Pro-apoptotic or pro-survival activity of viral proteins.

Viral Protein	Virus	Tested Cell Lines	Function	Reference
Core	DENV	HepG2	Pro-apoptotic	[30]
	WNV	BHK	Pro-apoptotic	[31]
		A549, HEL/18	Pro-survival	[32]
M	DENV, JEV, WNV, Yellow fever virus (YFV)	HeLa, HepG2, COS-7	Pro-apoptotic	[33]
E	Langat virus	Vero, Neuro-2a	Pro-apoptotic	[34]
	DENV	Progenitor cells of megakaryocytes	Pro-apoptotic	[35]
NS2A	WNV	A549, L929, Vero	Pro-apoptotic	[36,37]
NS2B and NS3	JEV, DENV, WNV	TE671, BHK	Pro-apoptotic	[38]

**Table 2:** Pro-apoptotic or pro-survival activity of flavivirus infection.

Virus	Tested Cell	Function	Reference
DENV	Neuro 2a	Pro-apoptotic	[42]
DENV	HUVEC EA.hy926	Pro-apoptotic	[43]
DENV	HMEC-1	Pro-apoptotic	[44]
DENV	HepG2	Pro-apoptotic	[45,46,47]
DENV	Hep3B	Pro-apoptotic	[47]
DENV	Monocyte derived dendritic cells (Mo-DC)	Pro-apoptotic	[48,49]
DENV	Huh7, BHK, Vero	Pro-apoptotic	[50]
WNV	Neuro 2a, K562	Pro-apoptotic	[51]
WNV	ES cells derived neuron	Pro-apoptotic	[52]
JEV, DENV	N18, A549, BHK	Pro-survival	[53]
WNV	SK-N-MC, MEF, HEK293T Primary rat hippocampal neuron	Pro-apoptotic	[55]
DENV	2fTGH, MEF	Pro-apoptotic	[56]
JEV	BHK	Pro-apoptotic	[57]
JEV	Neuro 2a	Pro-apoptotic	[58]
WNV	MEF	Pro-apoptotic	[59]
